# β-Blocker Use and Clinical Outcomes in Patients With COPD Following Acute Myocardial Infarction

**DOI:** 10.1001/jamanetworkopen.2024.7535

**Published:** 2024-05-21

**Authors:** David C. LaFon, Erika S. Helgeson, Sarah Lindberg, Helen Voelker, Surya P. Bhatt, Richard Casaburi, Steven J. Cassady, John Connett, Gerard J. Criner, Umur Hatipoglu, David A. Kaminsky, Ken M. Kunisaki, Stephen C. Lazarus, Charlene E. McEvoy, Robert M. Reed, Frank C. Sciurba, William Stringer, Mark T. Dransfield

**Affiliations:** 1 Division of Pulmonary, Allergy and Critical Care Medicine, Heersink School of Medicine, The University of Alabama at Birmingham; 2 UAB Lung Health Center, Heersink School of Medicine, The University of Alabama at Birmingham; 3 Division of Biostatistics and Health Data Science, University of Minnesota, Minneapolis; 4 Lundquist Institute for Biomedical Innovation, Harbor–UCLA Medical Center, Torrance, California; 5 Division of Pulmonary and Critical Care Medicine, University of Maryland, Baltimore; 6 Thoracic Medicine and Surgery, Lewis Katz School of Medicine at Temple University, Philadelphia, Pennsylvania; 7 Cleveland Clinic Lerner College of Medicine, Case Western Reserve University School of Medicine, Cleveland, Ohio; 8 Pulmonary and Critical Care Medicine, University of Vermont, Burlington; 9 Minneapolis VA Health Care System, Minneapolis, Minnesota; 10 Division of Pulmonary and Critical Care Medicine, University of California San Francisco; 11 Cardiovascular Research Institute, University of California San Francisco; 12 Health Partners Institute, Minneapolis, Minnesota; 13 Division of Pulmonary and Critical Care Medicine, University of Pittsburgh, Pittsburgh, Pennsylvania; 14 Birmingham VA Medical Center, Birmingham, Alabama

## Abstract

**Importance:**

While β-blockers are associated with decreased mortality in cardiovascular disease (CVD), exacerbation-prone patients with chronic obstructive pulmonary disease (COPD) who received metoprolol in the Beta-Blockers for the Prevention of Acute Exacerbations of Chronic Obstructive Pulmonary Disease (BLOCK-COPD) trial experienced increased risk of exacerbations requiring hospitalization. However, the study excluded individuals with established indications for the drug, raising questions about the overall risk and benefit in patients with COPD following acute myocardial infarction (AMI).

**Objective:**

To investigate whether β-blocker prescription at hospital discharge is associated with increased risk of mortality or adverse cardiopulmonary outcomes in patients with COPD and AMI.

**Design, Setting, and Participants:**

This prospective, longitudinal cohort study with 6 months of follow-up enrolled patients aged 35 years or older with COPD who underwent cardiac catheterization for AMI at 18 BLOCK-COPD network hospitals in the US from June 2020 through May 2022.

**Exposure:**

Prescription for any β-blocker at hospital discharge.

**Main Outcomes and Measures:**

The primary outcome was time to the composite outcome of death or all-cause hospitalization or revascularization. Secondary outcomes included death, hospitalization, or revascularization for CVD events, death or hospitalization for COPD or respiratory events, and treatment for COPD exacerbations.

**Results:**

Among 3531 patients who underwent cardiac catheterization for AMI, prevalence of COPD was 17.1% (95% CI, 15.8%-18.4%). Of 579 total patients with COPD and AMI, 502 (86.7%) were prescribed a β-blocker at discharge. Among the 562 patients with COPD included in the final analysis, median age was 70.0 years (range, 38.0-94.0 years) and 329 (58.5%) were male; 553 of the 579 patients (95.5%) had follow-up information. Among those discharged with β-blockers, there was no increased risk of the primary end point of all-cause mortality, revascularization, or hospitalization (hazard ratio [HR], 1.01; 95% CI, 0.66-1.54; *P* = .96) or of cardiovascular events (HR, 1.11; 95% CI, 0.65-1.92; *P* = .69), COPD-related or respiratory events (HR, 0.75; 95% CI, 0.34-1.66; *P* = .48), or treatment for COPD exacerbations (rate ratio, 1.01; 95% CI, 0.53-1.91; *P* = .98).

**Conclusions and Relevance:**

In this cohort study, β-blocker prescription at hospital discharge was not associated with increased risk of adverse outcomes in patients with COPD and AMI. These findings support use of β-blockers in patients with COPD and recent AMI.

## Key Points

**Question:**

In patients with chronic obstructive pulmonary disease (COPD) who are hospitalized for acute myocardial infarction, is β-blocker prescription at hospital discharge associated with increased risk of death or adverse respiratory or cardiovascular outcomes?

**Findings:**

In this cohort study of 3531 individuals with acute myocardial infarction, among 553 with COPD, β-blocker prescription at hospital discharge was not associated with increased risk of mortality or adverse cardiopulmonary outcomes.

**Meaning:**

The study did not find evidence that β-blockers are associated with increased risk of death or adverse respiratory or cardiovascular outcomes in patients with COPD following acute myocardial infarction.

## Teaser

This cohort study investigates whether a β-blocker prescription at hospital discharge is associated with mortality or adverse cardiopulmonary events among patients with chronic obstructive pulmonary disease hospitalized for acute myocardial infarction.

## Introduction

Chronic obstructive pulmonary disease (COPD) is the third leading cause of mortality worldwide,^1^ and cardiovascular disease (CVD) accounts for a large proportion of deaths in patients with COPD.^2,3^ Randomized clinical trials have demonstrated that β-blockers reduce the risk of death and reinfarction after acute myocardial infarction (AMI),^4,5^ and observational studies also suggest a potential benefit of β-blockers in patients with COPD even in the absence of a cardiac indication to receive the drug.^6,7,8,9,10^ The Beta-Blockers for the Prevention of Acute Exacerbations of Chronic Obstructive Pulmonary Disease (BLOCK-COPD) study was a multicenter, double-blind, placebo-controlled randomized clinical trial investigating the effect of metoprolol on exacerbation risk in COPD.^11^ The study examined the hypothesis that metoprolol would reduce exacerbation risk without an increase in adverse events. It was stopped early due to futility and safety concerns after an interim analysis indicated no difference in risk of any exacerbation but found a higher incidence of exacerbations requiring hospitalization in those assigned to receive metoprolol. These results suggested that β-blockers should not be prescribed to patients who have COPD with high exacerbation risk without a clear indication (eg, AMI, congestive heart failure). Notably, the clinical trials that demonstrated the cardiovascular benefits of β-blockers excluded patients with COPD,^4,12,13^ and it is therefore unknown whether the benefits of β-blockers following AMI extend to individuals with COPD. Prior observational studies of this question were affected by confounding due to historical avoidance of β-blockers in patients with more severe COPD^14,15^ and were also limited by a retrospective design, inadequate characterization of included patients with COPD, and incomplete follow-up data for respiratory events including COPD exacerbations. This knowledge gap provided the impetus for a contemporary study of the potential risks of β-blockers in patients with COPD who experience AMI, with robust characterization of participants and close longitudinal follow-up for cardiovascular and respiratory events.

We therefore performed a prospective, multicenter cohort study at sites in the BLOCK-COPD network to investigate differences in outcomes among patients with COPD following hospitalization for AMI who were discharged with vs without a β-blocker. The objectives of our study were to (1) determine the prevalence of COPD and disease-specific characteristics in patients admitted for AMI and (2) to investigate whether prescription for any β-blocker at hospital discharge was associated with higher risk of adverse cardiopulmonary outcomes.

## Methods

### Enrollment and Study Procedures

**Figure 1.**
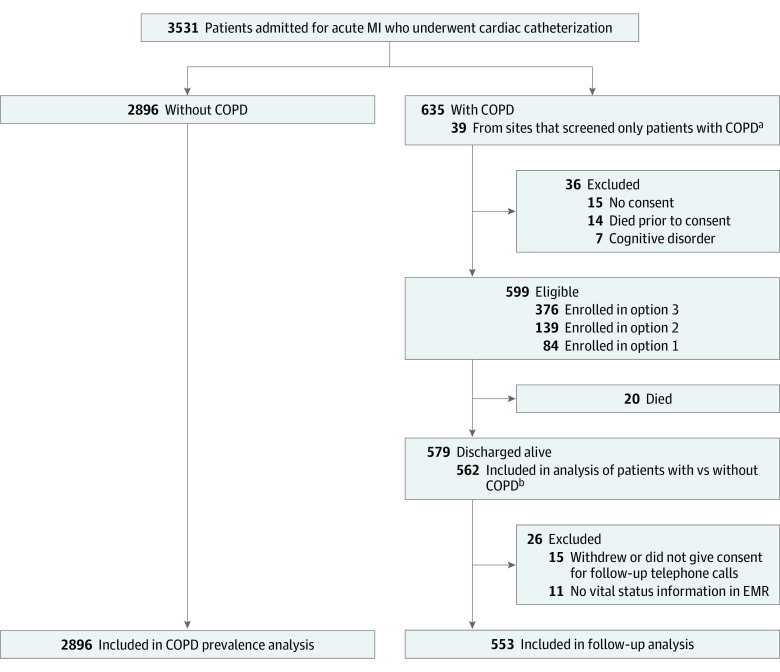
Screening, Enrollment, and Follow-Up Option 1 was an in-person visit during hospitalization with follow-up telephone calls and electronic medical record (EMR) review at 3 and 6 months; option 2 was EMR review during hospitalization and telephone calls and EMR review at 3 and 6 months; and option 3 was EMR review during hospitalization and follow-up EMR review at 3 and 6 months. COPD indicates chronic obstructive pulmonary disease; MI, myocardial infarction. ^a^Excluded from COPD prevalence estimate. ^b^Excluded from 2 sites that screened only patients with COPD.

We performed a prospective, longitudinal cohort study at 18 hospitals in the BLOCK-COPD network from June 2020 through May 2022. Study-related procedures were approved by the institutional review boards at each participating site. Patients aged 35 years or older with self-reported or physician-documented COPD who were admitted to the hospital with AMI and underwent left heart catheterization were identified via electronic medical record (EMR) review as eligible for participation (Figure 1). Basic demographic information was collected from patients with AMI but without COPD without further participation in the study. Due to limited access to hospitalized patients during the COVID-19 pandemic, 3 options for enrollment and follow-up were available (Figure 1). Option 1 involved an in-person visit during hospitalization, with follow-up telephone calls and EMR review at 3 and 6 months. In option 2, EMR review was performed during hospitalization, with postdischarge telephone consent and telephone calls and EMR review at 3 and 6 months. Finally, option 3, which was only used if local guidance did not allow for option 1 or 2, involved EMR review during hospitalization and follow-up EMR review at 3 and 6 months. Informed consent was obtained from participants with COPD enrolled via options 1 (written consent) and 2 (consent by telephone) in the prospective cohort, and a waiver of consent was obtained for participants in option 3 who were evaluated solely by EMR review because the study was considered low risk. This study followed the Strengthening the Reporting of Observational Studies in Epidemiology (STROBE) reporting guideline.

Initial EMR review and in-person visits included documentation of demographic information, smoking status, comorbidities, medication history, supplemental oxygen use, exacerbation history, results of pulmonary function tests (if available), and details of hospitalization. Follow-up telephone calls and EMR reviews at 3 and 6 months included assessment of vital status, date and cause of death, interval history of hospitalization and collection of relevant medical records, and interval history of steroid and/or antibiotic treatment for COPD exacerbation. Full details regarding BLOCK-COPD network sites, recruitment procedures, and full inclusion and exclusion criteria are available in the eMethods in Supplement 1.

Study investigators (D.C.L., M.T.D.) reviewed medical records and discharge summaries from hospitalization and revascularization events that occurred after the initial hospitalization. Events were categorized as related to heart failure, nonfatal MI, unstable angina, stroke, other CVD-related events, COPD, respiratory, or other. Race and ethnicity, included because rates of COPD diagnosis and disease outcomes may differ between groups, were obtained from the EMR within fixed categories based on the National Institutes of Health Diversity Program. Race categories included Black or African American, White, and other (American Indian or Alaska Native, Asian, Native Hawaiian or Pacific Islander, and multiracial). Ethnicity categories, collected only for patients with COPD, were Hispanic or Latino and not Hispanic or Latino.

### Outcomes

The primary outcome was time to the composite outcome of death or all-cause hospitalization or revascularization. Secondary outcomes included time to first CVD-related event, time to first COPD or respiratory event, rate of nonfatal events, rate of nonfatal CVD-related events, rate of nonfatal COPD or respiratory events, and rates of treatment with steroids and/or antibiotics for exacerbations.

Cardiovascular disease–related events included CVD-related death, revascularizations, and hospitalizations classified as heart failure, nonfatal MI, stroke, unstable angina, or other CVD events; COPD or respiratory events included COPD- or respiratory-related deaths or hospitalizations. Rates of treatment with steroids and/or antibiotics were determined from follow-up assessments, which documented how many times participants had received courses of oral corticosteroids or antibiotics for a respiratory condition or COPD since the last study contact.

### Sample Size

We estimated the need to recruit 571 patients with COPD and MI based on the following assumptions: between 45% and 65% of patients discharged with β-blockers, cumulative risk of death or rehospitalization at 6 months of 25% in patients prescribed β-blockers and 37.5% in patients not prescribed β-blockers,^16,17^ type I error of 5%, power of 80%, and in-hospital mortality of 12.5%.^18^ Full details on the sample size estimation are provided in the protocol (eAppendix in Supplement 1).

### Statistical Analysis

The prevalence estimate of COPD among those with MI was calculated as a simple fraction with an exact (Clopper-Pearson) 95% CI. Characteristics of individuals with or without COPD and those with AMI and COPD discharged with or without β-blockers were compared using Wilcoxon rank sum tests or χ^2^ tests unless otherwise noted. Kaplan-Meier curves and Cox proportional hazards regression models were used to evaluate time-to-event outcomes. Results for tests of proportional hazards assumption are provided in eTable 1 in Supplement 1. Annualized rates of nonfatal events were evaluated using negative binomial regression models.

The primary analysis used inverse probability of treatment weighting (IPTW) models to reduce confounding. Clinically important characteristics (age, sex, percutaneous coronary intervention during admission, ejection fraction, and number of courses of systemic corticosteroids and/or antibiotics for exacerbations in the prior year) and variables with a standardized mean difference greater than 0.10 between groups (eTable 2 in Supplement 1) were incorporated into a logistic regression model to create the propensity score for the inverse probability weights. Secondary analyses included unadjusted models and models adjusted for race, sex, age, smoking history, hospitalization for respiratory episodes in the previous year, prescription for home supplemental oxygen use, ejection fraction less than 40%, diagnosis of ST-elevation MI or non–ST-elevation MI for current admission, and asthma and stratified by clinic (for Cox proportional hazards regression models). Missing data were imputed using multivariate imputation by chained equations with predictive mean matching. Analyses were performed using R, version 4.32.1 (R Project for Statistical Computing). All hypothesis tests were 2-sided. *P* < .05 was considered statistically significant. No adjustments for multiplicity were performed, so the secondary analyses should be interpreted as exploratory. Additional details about the statistical methods are provided in the eMethods in Supplement 1.

## Results

The prevalence estimate of COPD among 3531 patients with AMI who underwent cardiac catheterization was 17.1% (95% CI, 15.8%-18.4%). A total of 562 with COPD (233 [41.5%] female; 329 [58.5%] male) and 2896 without COPD (949 of 2895 [32.8%] female; 1946 of 2895 [67.2%] male) were included in the analysis. Among those with COPD, 82 of 540 (15.2%) were Black or African American; 441 of 540 (81.7%), White; and 17 of 540 (3.1%), other race; among those without COPD, 537 of 2879 (18.7%) were Black or African American; 1920 of 2879 (66.7%), White; and 422 of 2879 (14.7%), other race. Characteristics of patients with and without COPD are shown in Table 1. Patients with COPD were older (median age, 70.0 years [range, 38.0-94.0 years] vs 65.0 years [range, 19.0-97.0 years]; *P* < .001) and were more likely to be female (*P* < .001), current or former smokers (521 [92.7%] vs 1534 [53.0%]; *P* < .001 for overall comparison), and White (*P* < .001 for overall comparison). Numerous comorbidities were more prevalent among patients with COPD, including hypertension, hyperlipidemia, coronary artery disease, heart failure, percutaneous coronary intervention, coronary artery bypass grafting, peripheral vascular disease, stroke, cirrhosis, asthma, obstructive sleep apnea, end-stage kidney disease, depression, anxiety, and cancer (Table 1).

**Table 1.** Comparison of Demographics and Medical History Between Individuals With and Without COPD Enrolled in the Study

Characteristic	Participants, No./total No. (%)		*P* value
	COPD (n = 562)	No COPD (n = 2896)	
Age, median (range), y	70.0 (38.0-94.0)	65.0 (19.0-97.0)	<.001
Sex			
Female	233/562 (41.5)	949/2895 (32.8)	<.001
Male	329/562 (58.5)	1946/2895 (67.2)	
Race^a^			
Black or African American	82/540 (15.2)	537/2879 (18.7)	<.001
White	441/540 (81.7)	1920/2879 (66.7)	
Other^b^	17/540 (3.1)	422/2879 (14.7)	
Smoking status			
Current smoker	206/562 (36.7)	609/2892 (21.1)	<.001
Former smoker	315/562 (56.0)	925/2892 (32.0)	
Never smoker	41/562 (7.3)	1358/2892 (47.0)	
Medical history			
Hypertension	508/562 (90.4)	2176/2894 (75.2)	<.001
Hyperlipidemia	456/562 (81.1)	1827/2894 (63.1)	<.001
Coronary artery disease	380/562 (67.6)	1248/2894 (43.1)	<.001
Myocardial infarction	274/562 (48.8)	767/2894 (26.5)	<.001
Heart failure	206/562 (36.7)	518/2894 (17.9)	<.001
Percutaneous coronary intervention	282/562 (50.2)	854/2894 (29.5)	<.001
Coronary artery bypass grafting	114/562 (20.3)	334/2894 (11.5)	<.001
Peripheral vascular disease	136/562 (24.2)	213/2894 (7.4)	<.001
Stroke	79/562 (14.1)	208/2894 (7.2)	<.001
Diabetes	234/562 (41.6)	1135/2894 (39.2)	.31
Cirrhosis	27/562 (4.8)	36/2894 (1.2)	<.001
Asthma	97/562 (17.3)	193/2894 (6.7)	<.001
Obstructive sleep apnea	138/562 (24.6)	346/2894 (12.0)	<.001
End-stage kidney disease	42/562 (7.5)	146/2894 (5.0)	.03
Depression	141/562 (25.1)	387/2893 (13.4)	<.001
Anxiety	142/562 (25.3)	324/2894 (11.2)	<.001
Cancer	123/562 (21.9)	394/2894 (13.6)	<.001
Organ transplantation	10/562 (1.8)	41/2894 (1.4)	.64

Abbreviation: COPD, chronic obstructive pulmonary disease.

^a^ Obtained from the electronic medical record.

^b^ Other included American Indian or Alaska Native, Asian, Native Hawaiian or Pacific Islander, and multiracial.

The overall in-hospital mortality rate among participants with COPD was 3.3% (20 of 599). Of the 579 patients with COPD who were discharged after AMI, 502 (86.7%) were prescribed a β-blocker and 77 (13.3%) were discharged without a β-blocker (Table 2). Those discharged with a β-blocker were more likely to have a history of coronary artery disease and less likely to have a history of stroke or anxiety. They also had lower rates of cardiogenic shock and shorter intensive care unit length of stay. Percentage of predicted postbronchodilator forced expiratory volume in 1 second, long-term oxygen use, prior COPD exacerbations, and use of oral corticosteroids in the past year did not differ between those discharged with a β-blocker vs without a β-blocker (standardized mean differences for comparisons are shown in eTable 2 in Supplement 1).

**Table 2.** Comparison of Demographics, Medical History, and Clinical Characteristics Between Individuals With COPD Who Were and Were Not Discharged With a Prescription for a β-Blocker

Characteristic	Participants^a^		*P* value
	Not discharged with β-blocker (n = 77)	Discharged with β-blocker (n = 502)^b^	
Demographics			
Age, median (range), y	68.0 (42.0-93.0)	70.0 (38.0-94.0)	.42
Sex			
Female	35 (45.5)	208 (41.4)	.59
Male	42 (54.5)	294 (58.6)	
Race^c^			
Black or African American	11/73 (15.1)	68/484 (14.0)	.92^e^
White	61/73 (83.6)	403/484 (83.3)	
Other^d^	1/73 (1.4)	13/484 (2.7)	
Ethnicity^c^			
Hispanic or Latino	1/76 (1.3)	13/496 (2.6)	.71^e^
Not Hispanic or Latino	75/76 (98.7)	483/496 (97.4)	
BMI, median (range)	27.0 (15.5-59.2)	28.0 (15.1-63.0)	.10
Smoking status			
Current	24 (31.2)	188 (37.5)	.51
Former	46 (59.7)	279 (55.6)	
Never	7 (9.1)	35 (7.0)	
Medical history			
Hypertension	67 (87.0)	454 (90.4)	.47
Hyperlipidemia	61 (79.2)	411 (81.9)	.69
Coronary artery disease	41 (53.2)	344 (68.5)	.01
Myocardial infarction	34 (44.2)	243 (48.4)	.57
Heart failure	25 (32.5)	189 (37.6)	.45
Percutaneous coronary intervention	30 (39.0)	255 (50.8)	.07
Coronary artery bypass graft	15 (19.5)	103 (20.5)	.95
Peripheral vascular disease	16 (20.8)	120 (23.9)	.65
Stroke	17 (22.1)	63 (12.5)	.04
Diabetes	32 (41.6)	205 (40.8)	≥.99
Cirrhosis	3 (3.9)	23 (4.6)	≥.99^e^
Asthma	17 (22.1)	87 (17.3)	.39
Obstructive sleep apnea	21 (27.3)	122 (24.3)	.67
End-stage kidney disease	5 (6.5)	33 (6.6)	≥.99
Depression	27 (35.1)	123 (24.5)	.07
Anxiety	32 (41.6)	121 (24.1)	.002
Cancer	14 (18.2)	110 (21.9)	.55
Organ transplantation	3 (3.9)	7 (1.4)	.14^e^
Treatments and characteristics during admission			
Thrombolytics	5 (6.5)	52 (10.4)	.39
Percutaneous coronary intervention	45 (58.4)	312 (62.2)	.62
Coronary artery bypass graft	4 (5.2)	64 (12.7)	.08
Experienced cardiogenic shock	16 (20.8)	47 (9.4)	.005
Diagnosis for current admission			
ST-elevation myocardial infarction	17 (22.1)	120 (23.9)	.84
Non–ST-elevation myocardial infarction	60 (77.9)	382 (76.1)	
Ejection fraction, median (range), %	55.0 (10.0-80.0)^f^	50.0 (10.0-77.0)^g^	.31
Admitted to ICU	28 (36.4)	187 (37.3)	.98
Time in ICU, median (range), d	4.5 (1.0-32.0)	2.0 (1.0-56.0)	.003
Intubated	11 (14.3)	85 (16.9)	.68
Discharge disposition			
Home	65 (84.4)	436 (86.9)	.52
Other	3 (3.9)	10 (2.0)	
Rehabilitation, long-term acute care, nursing home, or skilled nursing facility	9 (11.7)	56 (11.2)	
Time in hospital, median (range), d	3.2 (0.0-368.2)	4.1 (0.5-60.9)	.64
Discharged in hospice	3 (3.9)	9 (1.8)	.44
COPD characteristics			
Postbronchodilator FEV_1_, median (range), L	1.6 (0.7-2.7)^h^	1.7 (0.5-3.4)^i^	.39
Predicted postbronchodilator FEV_1_, median (range), %	51.5 (26.0-85.0)^h^	67.0 (24.0-105.0)^i^	.16
Prescription for home oxygen	16 (20.8)	78 (15.5)	.32
Exacerbation information			
Received a course of systemic corticosteroids and/or antibiotics in past year	21/77 (27.3)	111/502 (22.1)	.39
Courses, median (range), No.	1.0 (1.0-4.0)	1.0 (1.0-10.0)	.07
Respiratory episodes requiring care in the emergency department	11/21 (52.4)	69/111 (62.2)	.55
Respiratory episodes leading to hospitalization	10/21 (47.6)	56/111 (50.5)	≥.99
Hospitalizations, median (range), No.	1.0 (1.0-4.0)	1.0 (1.0-15.0)	.77
Respiratory episodes requiring intubation	0	4/111 (3.6)	≥.99^e^

Abbreviations: BMI, body mass index (calculated as weight in kilograms divided by height in meters squared); COPD, chronic obstructive pulmonary disease; FEV_1_, forced expiratory volume in 1 second; ICU, intensive care unit.

^a^ Data are presented as number or number/total number (percentage) of participants unless otherwise indicated.

^b^ Type of β-blocker could be ascertained for 491 individuals prescribed β-blockers (97.8%). Of these individuals, 354 (72.1%) were prescribed cardioselective β-blockers and 137 (27.9%) were prescribed noncardioselective β-blockers.

^c^ Race and ethnicity data were obtained from the electronic medical record within fixed categories based on the National Institutes of Health Diversity Program.

^d^ Other includes Asian, American Indian or Alaska Native, Native Hawaiian or Pacific Islander, and multiracial.

^e^ Fisher exact test *P* value.

^f^ Among 76 participants.

^g^ Among 499 participants.

^h^ Among 14 participants.

^i^ Among 83 participants.

**Figure 2.**
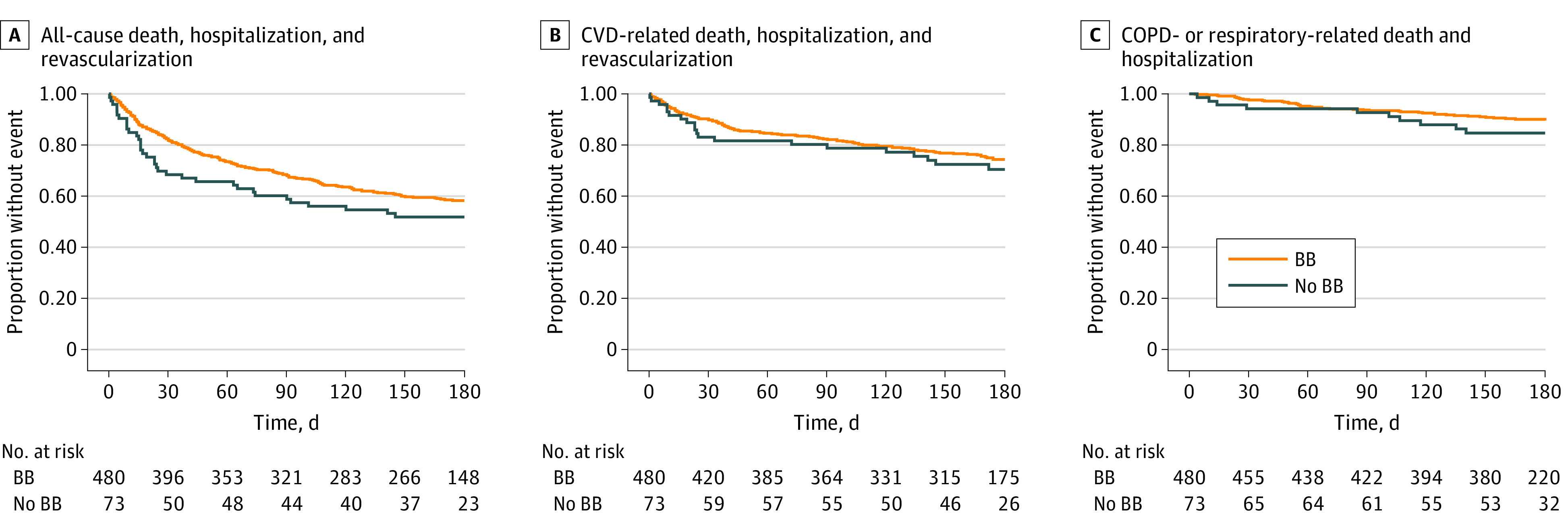
Kaplan-Meier Estimates of Primary and Secondary Outcomes BB indicates β-blocker; COPD, chronic obstructive pulmonary disease; and CVD, cardiovascular disease.

A total of 553 of the participants with COPD discharged after AMI (95.5%) had follow-up information and were included in the outcomes analysis (Figure 1). The median length of follow-up was 179 days (IQR, 170-180 days). Thirty-five of the 73 individuals (47.9%) not prescribed a β-blocker and 195 of 480 (40.6%) who were prescribed a β-blocker experienced the composite primary end point of all-cause mortality, hospitalization, or revascularization (eTable 3 in Supplement 1). There was not a significant difference in risk of the primary outcome between individuals with COPD who were discharged with a β-blocker vs without a β-blocker (unadjusted hazard ratio [HR], 0.77 [95% CI, 0.54-1.10]; *P* = .15; IPTW HR, 1.01 [95% CI, 0.66-1.54]; *P* = .96) (Table 3 and Figure 2A). Overall rates of hospitalization or revascularization events did not significantly differ between the groups (IPTW rate ratio [RR], 1.03 [95% CI, 0.66-1.60]; *P* = .91) (Table 3).

**Table 3.** Study Outcomes

Outcome	Unadjusted model		Adjusted model^a^		IPTW model	
	Result (95% CI)^b^	*P* value	Result (95% CI)^b^	*P* value	Result (95% CI)^b^	*P* value
Primary						
Death, hospitalization, or revascularization	0.77 (0.54-1.10)	.15	0.85 (0.59-1.24)	.40	1.01 (0.66-1.54)	.96
Secondary						
CVD death or CVD-related hospitalization or revascularization	0.84 (0.52-1.35)	.46	0.99 (0.60-1.65)	.98	1.11 (0.65-1.92)	.69
Respiratory- or COPD-related death or hospitalization	0.65 (0.33-1.28)	.21	0.79 (0.37-1.67)	.53	0.75 (0.34-1.66)	.48
Nonfatal event						
Treatment with steroids and/or antibiotics for exacerbations	0.87 (0.43-1.78)	.70	0.89 (0.46-1.72)	.73	1.01 (0.53-1.91)	.98
Overall hospitalization or revascularization	0.82 (0.53-1.29)	.39	0.89 (0.59-1.34)	.57	1.03 (0.66-1.60)	.91
Hospitalization or revascularization for CVD events	1.18 (0.66-2.11)	.58	1.35 (0.76-2.38)	.31	1.51 (0.89-2.59)	.13
COPD or respiratory events	0.69 (0.32-1.51)	.35	0.76 (0.36-1.61)	.48	0.76 (0.36-1.60)	.47

Abbreviations: COPD, chronic obstructive pulmonary disease; CVD, cardiovascular disease; IPTW, inverse probability of treatment weighting.

^a^ Model adjusted for race, sex, age, smoking history, hospitalization for respiratory episodes in previous year, prescription for home supplemental oxygen use, ejection fraction less than 40%, diagnosis of ST-elevation myocardial infarction or non–ST-elevation myocardial infarction for current admission, and asthma. Cox proportional hazards regression models were additionally stratified by clinic.

^b^ Primary and secondary outcomes were assessed using hazard ratios, and nonfatal events were assessed using rate ratios.

Among the 73 individuals who were not prescribed a β-blocker, 20 (27.4%) experienced the secondary outcome of CVD-related events (revascularization, CVD-related hospitalization, or CVD-related death) (eTable 3 in Supplement 1). Of the 480 who were prescribed a β-blocker, 114 (23.8%) experienced this end point. There were no significant associations between β-blocker prescription and time to a CVD-related event (IPTW HR, 1.11 [95% CI, 0.65-1.92]; *P* = .69) or rate of CVD-related hospitalization or revascularization (IPTW RR, 1.51 [95% CI, 0.89-2.59]; *P* = .13) (Table 3 and Figure 2B).

Ten of 73 participants (13.7%) discharged without a β-blocker and 45 of 480 (9.4%) discharged with a β-blocker experienced the secondary outcome of a COPD- or respiratory-related event (which included hospitalization or death) (eTable 3 in Supplement 1). We did not find a difference between β-blocker prescription and time to respiratory- or COPD-related death or hospitalization (IPTW HR, 0.75 [95% CI, 0.34-1.66]; *P* = .48) or rate of respiratory or COPD-related hospitalizations (IPTW RR, 0.76 [95% CI, 0.36-1.60]; *P* = .47) (Table 3 and Figure 2C). We did not find a significant difference in rate of treatment with steroids and/or antibiotics for exacerbations between the groups (IPTW RR, 1.01 [95% CI, 0.53-1.91]; *P* = .98) (Table 3).

## Discussion

To our knowledge, this study is the first to prospectively investigate the effects of β-blockers in a cohort of patients with COPD following AMI. Notably, we found a high rate of β-blocker prescription at discharge among patients with COPD in this cohort. This stands in contrast to earlier studies that described a high rate of withholding β-blockers in patients with COPD due to concerns regarding adverse effects.^14,19^ We did not find evidence of increased risk of all-cause mortality, hospitalizations, or revascularizations among those discharged with β-blockers. We also did not find increased risk of COPD exacerbations or other events when restricted to respiratory-, COPD-, or CVD-related factors.

As expected, we observed differences in patient characteristics between those with and without COPD. There is increasing recognition of the importance of multimorbidity in patients with COPD.^20^ In addition to higher rates of CVD, we also observed greater prevalence of cirrhosis, end-stage kidney disease, obstructive sleep apnea, anxiety, and depression. These comorbidities have been associated with more severe respiratory symptoms and worse outcomes.^21^ Interestingly, patients with COPD who had anxiety were less likely to be discharged with a β-blocker. Those with a history of stroke were also less likely to be discharged with a β-blocker. It is possible that β-blocker intolerance and adverse effects (eg, dizziness, orthostatic hypotension, bradycardia, and depression risk) would be expected to be particularly problematic in this population. The lower rates of β-blocker prescription among those with cardiogenic shock and a longer intensive care unit length of stay were most likely related to hemodynamic instability and withholding of β-blockers in accordance with guidelines for management of cardiogenic shock.

Most patients with COPD who were hospitalized with AMI were discharged with a β-blocker. This suggests that COPD is not viewed as a contraindication to β-blocker use in patients with AMI. Likewise, we found no differences in COPD severity (as evaluated by spirometric lung function, long-term oxygen use, and previous exacerbations) between groups to suggest that β-blockers were avoided among those with more advanced disease.

The current study used broader inclusion criteria compared with the BLOCK-COPD trial, which used more stringent criteria for spirometric impairment, exacerbation history, and baseline vital signs (in addition to excluding individuals with a cardiovascular indication for β-blocker use).^11^ Despite enrollment of a broader COPD cohort, there was evidence of high morbidity following AMI. Our observed in-hospital mortality rate was 3.3% compared with 4.6% in a prior large, multicenter study.^22^ Substantial proportions of those discharged with and without a β-blocker (40.6% and 47.9%, respectively) experienced the primary outcome of death, reinfarction, or rehospitalization. With access to detailed medical records, we were able to determine primary causes for subsequent hospitalization events and did not find evidence of increased hospitalization for CVD, respiratory, or COPD events in those who received β-blockers. Similarly, we did not observe increased incidence of the secondary end point of treatment with antibiotics and/or systemic corticosteroids for COPD exacerbations among those discharged with β-blockers, which would include less severe exacerbations that may have been managed in an outpatient setting and therefore not captured as hospitalization events. Our finding of a lack of an increase in respiratory events in a cohort that was at risk for adverse outcomes may help address safety concerns in patients with COPD and a cardiovascular indication for β-blocker use.

### Strengths and Limitations

Strengths of our study include enrollment of participants from multiple sites; detailed, prospective data collection; characterization of COPD-specific variables and outcomes; and a rigorous statistical analysis that included both multivariable adjusted models and IPTW models to reduce confounding. Limitations of our study include the relatively small number of patients who were discharged without β-blockers, which likely limited the power of our study to detect modest differences in outcomes. Our inclusion criteria used patient- or practitioner-reported (rather than spirometrically confirmed) COPD, and possible misclassification could have affected the results; however, our diagnostic criteria reflect prescribing scenarios most frequently encountered in clinical practice. We also lacked detailed information for patients without COPD that may have enabled comparison of rates of β-blocker use with participants with COPD in our study. To reduce the chances of enrolling patients who had a systemic illness resulting in a supply-demand mismatch (ie, type 2 non–ST elevation MI) rather than AMI caused by an acute coronary occlusion, we only included patients who underwent cardiac catheterization. This might have led to the exclusion of those with advanced age or more severe disease or comorbidities or others who received noninvasive management for AMI. It is also possible that subsequent hospitalization events at facilities outside the BLOCK-COPD network may not have been captured if study staff were not contacted via telephone with notification of the event.

## Conclusions

In this cohort study, β-blocker prescription at hospital discharge was not associated with increased risk of adverse outcomes in patients with COPD and AMI. The results add valuable context to the findings of the BLOCK-COPD trial,^11^ which observed increased risk of hospitalization for exacerbations among individuals with COPD with high exacerbation risk who received metoprolol. Although we cannot rule out potential harm in selected patients, our findings support the continued use of β-blockers among patients with COPD who have AMI.

## Notes

Supplement 1.
eMethods
**eAppendix.** Detailed Study Protocol **eTable 1.** Assessment of the Proportional Hazards Assumption **eTable 2.** Standardized Mean Differences for Comparison of Demographics, Medical History, and Clinical Characteristics Between Individuals With COPD With Follow-Up Information Who Were and Were Not Discharged With a Prescription for β-Blockers **eTable 3.** Number of Individuals Who Experienced Each Event Type by β-Blocker Prescription at Discharge
eReferences

Supplement 2.
Data Sharing Statement
