# Supplementary material for: β-Blocker Use and Clinical Outcomes in Patients With COPD Following Acute Myocardial Infarction
Source: JAMA Netw Open. 2024 May 21;7(5):e247535. doi: 10.1001/jamanetworkopen.2024.7535 (PMC11109775; doi:10.1001/jamanetworkopen.2024.7535)
Supplement: Supplement 1. — eMethods eAppendix. Detailed Study Protocol eTable 1. Assessment of the Proportional Hazards Assumption eTable 2. Standardized Mean Differences for Comparison of Demographics, Medical History, and Clinical Characteristics Between Individuals With COPD With Follow-Up Information Who Were and Were Not Discharged With a Prescription for β-Blockers eTable 3. Number of Individuals Who Experienced Each Event Type by β-Blocker Prescription at Discharge eReferences [file jamanetwopen-e247535-s001.pdf]

Supplementary Online Content

LaFon DC, Helgeson ES, Lindberg S, et al. β-Blocker use and clinical outcomes in patients with COPD following acute myocardial infarction. *JAMA Netw Open*. 2024;7(5):e247535. doi:10.1001/jamanetworkopen.2024.7535

eMethods

eAppendix. Detailed Study Protocol

eTable 1. Assessment of the Proportional Hazards Assumption

eTable 2. Standardized Mean Differences for Comparison of Demographics, Medical History, and Clinical Characteristics Between Individuals With COPD With Follow-Up Information Who Were and Were Not Discharged With a Prescription for β-Blockers

eTable 3. Number of Individuals Who Experienced Each Event Type by β-Blocker Prescription at Discharge

eReferences

This supplementary material has been provided by the authors to give readers additional information about their work.

© 2024 LaFon DC et al. *JAMA Network Open*.

eMethods

The prevalence estimate of COPD among those with MI was calculated as a simple fraction with an exact (Clopper–Pearson) confidence interval based on all individuals admitted to the hospital regardless of whether they meant inclusion criteria for the study. Characteristics of individuals with or without COPD, and those with acute MI and COPD discharged with or without beta blockers were compared using Wilcoxon-rank sum test or chi-square tests unless otherwise noted.

Kaplan-Meier curves and Cox proportional hazards models were used to compare time to death or all-cause hospitalization or revascularization; time to CVD-related death, hospitalization, or revascularization; and time to respiratory or COPD-related death or hospitalization between patients with AMI and COPD who were and were not discharged alive on beta-blockers. The primary analysis used Cox proportional hazard models with inverse probability of treatment weighting (IPTW) to reduce confounding. Clinically important characteristics (age, sex, PCI during admission, ejection fraction, number of courses of systemic corticosteroids and/or antibiotics for exacerbations in the prior year) and variables with a standardized mean difference between groups >0.10 (supplementary Table 1) were incorporated into a logistic regression model to create the propensity score for the inverse probability weights. Weights were truncated at the 95% percentile. Unadjusted cox proportional models and models adjusted for pre-specified covariates are also presented.

Annualized rates of all-cause hospitalization or revascularizations, CVD-related hospitalizations, COPD or respiratory related hospitalizations, and treatment with steroids or antibiotics for exacerbations were compared between patients with AMI and COPD who were discharged alive on beta-blockers to that of patients not discharged on beta-blockers using negative binomial regression models. Models included IPTW weighted models, unadjusted models, and models adjusted for pre-specified covariates.

Death, hospitalization, and revascularization events were censored at last known follow-up day or 6-months post-discharge. For time to CVD-related outcomes those who did not experience a CVD-related event and died due to non-CVD causes were censored at time of death. A similar procedure was used for COPD/respiratory outcomes. There was no censoring for steroid/antibiotic use for exacerbations since information could only be obtained from the month 3 and 6 follow-up forms. Adjusted models included race, sex, age, smoking history, hospitalization for respiratory episodes in previous year, prescription for home supplemental oxygen use, ejection fraction <40%, diagnosis of STEMI/non-STEMI for current admission, and asthma, and Cox-proportional hazards models were stratified by clinic. Missing values for ejection fraction and spirometry measures were imputed using multivariate imputation by chained equations with predictive mean matching. Auxiliary variables included in the imputation included age, sex, race, clinic, BMI, smoking status, medical history of high blood pressure, asthma, coronary artery disease, percutaneous coronary intervention, stroke, depression, anxiety, cancer, transplantation; thrombolytics, coronary artery bypass grafting, percutaneous coronary intervention, shock, ejection fraction, days in ICU, days in hospital, and diagnosis for admission; discharge disposition and discharged on hospice after admission; pre or post bronchodilator spirometry measures, FEV_1 (% predicted, FVC (% predicted), prescription for home oxygen; and number of courses of systemic corticosteroids and/or antibiotics, number of hospitalizations , and intubation in past year for respiratory episodes.

Results were pooled across the 30 imputations using Rubin's rules (1) for computing the total variance and weights were averaged across the imputations. Analyses were performed using R software version 4.32.1 (R Core Team) and the following packages: survival (3.5-5), survminer (0.4.9) mice (3.16.0), survey (4.2-1), sjstats (0.18.2), and tabelone (0.13.2), respectively. No adjustments for multiplicity were performed so the secondary analyses should be interpreted as exploratory.

© 2024 LaFon DC et al. *JAMA Network Open*.

BLOCK-COPD Network Hospitals:

1. New York Presbyterian/Weill Cornell Medical Center, New York, NY USA
2. Louisiana State University, New Orleans, LA USA
3. North Florida-South Georgia Veterans Health System, Gainesville, FL USA
4. Providence Medical Group, Spokane, WA USA
5. Harbor-UCLA Medical Center, Los Angeles, CA USA
6. Temple University, Philadelphia, PA USA
7. National Jewish Health, Denver, CO USA
8. University of Vermont, Burlington, VT USA
9. Minneapolis VA Health Care System, Minneapolis, MN USA
10. University of California San Francisco, San Francisco, CA USA
11. Mayo Clinic, Rochester, MN USA
12. Northwestern University, Chicago, IL USA
13. University of Michigan, Ann Arbor, MI USA
14. University of Alabama at Birmingham, Birmingham, AL USA
15. Health Partners Minnesota, Minneapolis, MN USA
16. University of Maryland, Baltimore, MD USA
17. Cleveland Clinic, Cleveland, OH USA
18. University of Pittsburgh, Pittsburgh, PA USA

© 2024 LaFon DC et al. *JAMA Network Open*.

eAppendix. Detailed Study Protocol:

An Observational Study of Beta-Blocker Use in Patients with Chronic Obstructive Pulmonary Disease (COPD) and Acute Myocardial Infarction

Version 4.0

May 20, 2020

1. Introduction

Chronic obstructive pulmonary disease (COPD) is the third leading cause of death worldwide and a major contributor to morbidity and mortality is comorbid cardiovascular disease. COPD is an independent risk factor for coronary artery disease and myocardial infarction (MI) is a common cause of death in those with the disease. Beta-blockers have been demonstrated to reduce the risk of death and reinfarction after MI and though observational studies suggest that this benefit extends to patients with COPD, this has not been definitively proven in randomized controlled trials. Results of the recently published randomized, placebo-controlled trial, Metoprolol for the Prevention of Acute Exacerbations of COPD (BLOCK COPD), demonstrate that beta-blockers do not reduce the risk of COPD exacerbations and increase the risk of exacerbations requiring hospitalization raising questions about the overall risk-benefit of the drugs in COPD patients, including those with class I indications such as MI. We propose a multicenter, observational study to examine the association between beta-blocker use and all-cause mortality and hospitalization following MI in patients with COPD.

2. Background

Results and Clinical Implications of the BLOCK COPD Trial

Chronic obstructive pulmonary disease (COPD) is the third leading cause of death worldwide and the majority of COPD-related morbidity, mortality and healthcare costs are driven by exacerbations, particularly those requiring hospitalization (1, 2). Possible explanations for the failure of existing treatments to control exacerbations adequately include the fact that events may be triggered or made more severe by underlying cardiovascular disease(3). Compared to age-matched controls, COPD is associated with up to a 5-fold risk of cardiovascular disease(4) which is a risk factor for exacerbation (5), hospitalization for exacerbation (6), in-hospital mortality (7, 8), and reduced survival (9, 10).

It is well established that beta-blockers reduce mortality in patients after myocardial infarction(11) and in the presence of heart failure.(12) Patients with COPD are often denied this class of medications, even in the presence of evidence based indications for their use, because of concerns about possible adverse effects on lung function.(13, 14) This practice pattern persists despite multiple observational studies suggesting beta-blockers benefit patients with COPD and comorbid cardiovascular disease with outcomes comparable to those observed in patients without COPD (13, 15, 16). Recent non-randomized studies also suggest that beta-blockers reduce the risk of exacerbations and mortality in patients with COPD, with or without known cardiac disease.(17-20) However, these observational data are subject to biases, precluding conclusions about cause and effect (21).

To address these issues, we investigated the effect of metoprolol on the risk of exacerbations in COPD patients at high risk for those events (ClinicalTrials.gov number: NCT02587351) (22, 23). We hypothesized that metoprolol would reduce the risk of exacerbations compared with placebo and would not adversely impact lung function, six-minute walk distance, dyspnea, or quality of life.

We randomized 532 participants with a mean (±SD) age of 65.0±7.8 years and FEV_1 of 41.1±16.3 % predicted. The study was stopped early due to futility for the primary endpoint and safety concerns. There was no difference in time to first exacerbation between groups and the hazard ratio (HR) for assignment to metoprolol was 1.05 (95%CI: 0.84-1.32; p=0.66). Metoprolol was associated with a higher risk of exacerbation requiring hospitalization (HR 1.91, 95%CI: 1.29-2.83). Patient reported possible beta-blocker side effects were similar between groups as was the overall rate of non-respiratory serious adverse events. During the treatment period, we observed 11 deaths in the metoprolol group and 5 in the placebo group.

© 2024 LaFon DC et al. *JAMA Network Open*.

The results of the study strongly indicate that patients with COPD and at risk for exacerbation should not be prescribed beta-blockers in the absence of an established indication for the drugs such as recent myocardial infarction or heart failure.

Unanswered Questions and the Rationale for the Proposed Study

As noted above, retrospective observational studies have suggested that the benefits of beta-blockers in patients with recent myocardial infarction and heart failure extend to those with COPD (13, 15, 19, 24), however, this has not been prospectively confirmed and randomized trials in those settings may be needed to assess overall risk-benefit. Though such trials are beyond the scope of the current award, we do have the opportunity to conduct a prospective, observational cohort study to examine cardiac and pulmonary outcomes in COPD patients hospitalized for acute myocardial infarction who are and are not treated with beta-blockers.

Several large randomized trials published in the 1980s demonstrated the clinical efficacy of beta-blockers following MI but COPD patients were largely excluded from these trials because of concerns about adverse respiratory effects. For example, the Metoprolol in Acute Myocardial Infarction (MIAMI) trial excluded patients with COPD who required steroids or beta-agonists (25). The Beta-Blocker Heart Attack Trial (BHAT) excluded patients with both asthma and COPD if they required therapy (24) and the Norwegian Timolol Study excluded all patients with COPD(11). As such, these data do not provide confidence that the benefits observed in patients without COPD extend to those with the disease, particularly those with more symptoms and requiring respiratory treatments.

A number of observational studies examining this issue have been conducted in an effort to provide evidence that can guide clinician decision making but these have been limited by their retrospective design, poor characterization of the COPD patients enrolled, and a lack of adequate follow-up data about pulmonary outcomes including COPD exacerbations.

Gottlieb et al. examined the medical records of more than 200,000 patients with MI as part of the Cooperative Cardiovascular Project (CCP) and found that though beta-blocker use was less common in those with underlying COPD (22% vs. 34%), the overall benefit of beta-blockers on mortality was comparable to that observed in those without COPD (Relative Risk (RR) 0.60; 95%CI, 0.57-0.63).(15) However, no information about the severity of the underlying lung disease was available and the possibility that healthier patients received beta-blockers cannot be excluded. A second study examining data from the CCP did stratify patients with COPD based on whether they were receiving beta-agonists or had been treated with oral steroids or admitted for COPD in the year prior to their MI.(24) In that analysis, COPD patients without these characteristics derived the same mortality benefit from beta-blockers as did patients without COPD (RR 0.85; 95%CI, 0.73-1.00) but no benefit was observed in those treated with beta-agonists or treated for acute symptoms in the year prior. A more recent population-based cohort study from the UK of 1063 patients with COPD and MI occurring between 2003 and 2008 found that treatment with beta-blockers in the hospital, or at the time of discharge, was associated with improved survival compared to no beta-blocker treatment(13). Though this analysis adjusted for COPD severity as assessed by treatment, symptoms and exacerbation history, a large number of patients were excluded as beta-blocker treatment was viewed as contraindicated by the treating physician, in some cases due to COPD. This precludes examination of outcomes in this likely more severely affected group.

The results of our proposed observational study would provide more robust and contemporary data regarding the overall risk-benefit of beta-blockers in COPD patients with acute MI and provide preliminary data about the feasibility of recruitment for a subsequent randomized trial.

3. Specific Aims

Specific Aim 1. To determine the prevalence of COPD in patients admitted to the hospital with an acute myocardial infarction (AMI) and to characterize the phenotypic expression and severity of their underlying lung disease.

Patients admitted to the hospital and who undergo cardiac catheterization for an AMI will be identified through the electronic medical record (EMR). Those with a diagnosis of COPD in the EMR will be offered participation in the study. Baseline characterization will include demographics, smoking history, prior history of exacerbations in the year before admission, supplemental oxygen use, respiratory and cardiac medication use, comorbidities including history of coronary artery disease, heart failure and ejection fraction, and pulmonary function data as available in the EMR.

© 2024 LaFon DC et al. *JAMA Network Open*.

Results of this Aim will provide data about the prevalence and clinical characteristics of COPD in the hospitalized population with AMI in our network. The Aim will also provide an estimate of the number of annual admissions for patients with COPD and AMI.

Specific Aim 2. To determine the association between beta-blocker use at discharge and cardiopulmonary outcomes in patients with COPD and AMI.

Patients with AMI and EMR-documented COPD will be followed, prospectively from the time of discharge using review of the electronic medical record and as local guidance permits, phone calls at 3 and 6 months. We will determine the associations between beta-blocker use at discharge and the risk for all-cause mortality, recurrent ischemic events, and hospitalization for COPD exacerbation adjusting for baseline characteristics and COPD severity.

4. **Study Design and Methods**

a) **Overview.** The study is a prospective, observational study of patients admitted to the BLOCK COPD network hospitals with acute AMI and COPD. Patients with will be identified via the EMR. Because of the current COVID-19 pandemic and the possibility of limited access to hospitalized patients, the study consists of three options for enrollment. Option 1 or 2 is preferable if local guidance permits.

Option 1: A total of 3 visits including 1 in person visit in the hospital and 2 follow up phone calls with EMR review at 3 and 6 months after discharge. Sites may consider alternatives to in person consent and data collection including by telephone or video conference.

Option 2: EMR review at the time of hospital admission followed by post-discharge telephone consent and 2 follow up phone calls with EMR review at 3 and 6 months.

Option 3: EMR review at the time of hospital admission and follow-up review of the EMR at 3 and 6 months after discharge.

b) **Participant selection and eligibility.** Subjects hospitalized and who undergo cardiac catheterization with AMI and have EMR-documented COPD will be eligible for participation. The age, race, gender, smoking status and comorbidities of patients with who undergo cardiac catheterization for AMI but have no EMR-documented diagnosis of COPD will be recorded but no other information will be collected on this population.

c) **Inclusion Criteria**

a. Willing and able to provide informed consent (applicable for Option 1 and 2 only)
b. Men and women age 35 or older
c. Admitted to hospital from the Emergency Department or by hospital to hospital transfer with a primary diagnosis of AMI
d. Undergo cardiac catheterization for AMI
e. EMR-documented COPD

d) **Exclusion Criteria**

a. Cognitive disorder that in the judgment of the investigator impairs understanding of the study objectives or assessments (applicable for Option 1 only)
b. Vulnerable populations, including prisoners and pregnant women

e) **Consent Process.** Patients admitted and who undergo cardiac catheterization for AMI will be identified via the EMR. If protocol Option 1 is being followed those with EMR-documented COPD will be approached for participation. They will be asked to provide written consent only after the objectives and risks of the study have been fully explained. Surrogate consent will not be permitted. The reasons for patients not being eligible for the study will be recorded. If protocol Option 2 is being

© 2024 LaFon DC et al. *JAMA Network Open*.

followed a waiver of informed consent will be obtained from local IRBs for the initial EMR review and then telephone consent will be obtained after discharge. If Option 3 is being followed a waiver of consent will be obtained.

f) **Study Visit Schedule and Assessments**

a. In-hospital visit and EMR review. All data elements will be obtained from the EMR except the self-reported COPD assessment which will be obtained during the hospitalization and after informed consent in protocol Option 1 and after discharge and phone consent in protocol option 2.
   i. Demographics to include age, gender, race and ethnicity, height, weight
   ii. Smoking status
   iii. COPD assessment - year of diagnosis, home use of supplemental oxygen, self-reported history of spirometry being conducted, number of hospitalizations for COPD exacerbations in the year prior to admission, number of courses of antibiotics and/or steroids for COPD exacerbations in the year prior to admission. For protocol Option 1 this will be collected via self-report utilizing a questionnaire during the hospitalization. For protocol Option 2, this information will be obtained after discharge and phone consent. For protocol Option 3 as much of this information as possible will be collected from review of the EMR.
   iv. Most recent forced expiratory volume (FEV1) and forced vital capacity (absolute and percent predicted) from EMR if documented in the 3 years prior to admission
   v. Comorbidity assessment to include cardiovascular disease and risk: history of hypertension, hyperlipidemia, coronary artery disease, prior MI, heart failure (ejection fraction prior to admission if available), percutaneous coronary intervention (PCI), coronary artery bypass grafting (CABG), peripheral vascular disease, stroke, diabetes mellitus, cirrhosis, asthma, obstructive sleep apnea, depression, anxiety, end-stage renal disease, cancer (type), organ transplantation (type)
   vi. Respiratory medication use on admission
   vii. Cardiovascular medication use on admission
   viii. Assessment of in hospital MI treatment: thrombolytics, PCI, CABG
   ix. Diagnosis of ST elevation MI (STEMI) or non-ST elevation MI (NSTEMI)
   x. Initial post MI ejection fraction from inpatient echocardiogram
   xi. Length of hospital stay
   xii. Length of ICU stay
   xiii. Need for intubation
   xiv. Cardiogenic shock during hospital stay
   xv. Discharge cardiovascular medications
   xvi. Discharge disposition

b. Phone calls (Option 1 only) and EMR review at 3 and 6 months
   i. Vital status
   ii. Date and cause of death if applicable
   iii. Interval history of hospitalization and primary diagnosis (all events will be captured but hospitalizations for COPD exacerbations, recurrent MI, revascularization, or heart failure will be specifically queried
   iv. Interval history of treatment with steroids or antibiotics for exacerbations of COPD
   v. Beta-blocker use and reason for discontinuation, if applicable Medical records will be collected for all admissions

g) Analytic and Statistical considerations.

a. Analysis plan for Aim 1. The primary objectives of Aim 1 are to determine the number of admissions per year for AMI at our participating institutions and the prevalence of COPD in that population. These prevalence estimates will be calculated as a simple fraction. We will

© 2024 LaFon DC et al. *JAMA Network Open*.

also compare the clinical characteristics of COPD patients discharged on a beta-blocker versus those not discharged on a beta-blocker using t-tests or chi-square tests (or Fisher's exact tests) as appropriate.

b. Analysis plan for Aim 2. For the primary analysis we will compare the risk of death or all-cause hospitalization at 6 months for patients with AMI and COPD who were discharged alive on beta-blockers to that of patients not discharged on beta-blockers. Secondary analyses will include comparisons of rates of hospitalization for COPD exacerbations, rates of hospitalization for recurrent MI or revascularization or heart failure, and rates of treatment with steroids and/or antibiotics for exacerbations. The primary analysis will be based on adjusted Kaplan–Meier survival curves that describe the probability remaining alive and out of hospital in those discharged versus not discharged on beta-blockers, while adjusting for the inverse probability of treatment weighting (IPTW) to reduce confounding. For all time-to-event analyses, the start time will be when the participant is discharged from the hospital. Participants will be censored at 6 months post-discharge if no event occurred. The comparison of clinical characteristics between treatment groups conducted in aim 1 will be used to inform the calculation of the IPTW. We will use the adjusted log-rank test to compare the two curves. As secondary analyses, we will use both IPTW and adjusted Cox proportional-hazards models to assess the association between the beta-blocker prescription and death or re-hospitalization. Adjusted models will also include the covariates of race, sex, baseline age, smoking status, number of hospitalizations for COPD during the previous year, use of supplemental oxygen, presence of ejection fraction <40% at discharge. Assuming adequate frequencies between groups, we will stratify the models by beta-blocker use at the time of admission, presence of asthma, STEMI vs. NSTEMI, and site. Rates of exacerbation will be analyzed by IPTW negative binomial regression models and will be used to compare annualized rates of hospitalization.

c. Sample size and power estimates. Prior data suggests that the prevalence of COPD in patients admitted with acute MI is between 13 and 25% depending on the population studied though these data have depended primarily on administrative coding (13, 16, 26-28). COPD is an independent risk factor for death following MI with estimates of in-hospital mortality of 12.5% versus 8.6% in those without COPD (28). Estimates for 1-year mortality for those with MI and COPD range from 14-25% versus 7-14% in those without COPD (29-33). Estimates of the benefit of beta-blockers in patients with COPD vary widely with studies demonstrating reductions in the risk of mortality between 9 and 50% (4, 13, 15, 16, 34). Data regarding the risk of re-hospitalization in COPD patients with MI and the association with beta-blocker use is scarce. However, the overall rate of readmission for recurrent MI, revascularization, heart failure, serious bleeding, as well as respiratory causes is 1.5-2 fold higher in those with COPD compared to those without the disease with a cumulative risk at 6 months between 25 and 45% (33, 35). Sample size calculations for the proposed study are therefore based on the following assumptions: The estimated sample size (number of patients with COPD and MI) assuming: Risk of death or re-hospitalization at 6 months in patients not prescribed beta blockers – between 32.5 to 37.5%; risk of death or re-hospitalization at 6 months in patients prescribed beta blockers – 25%; proportion of patients discharged on beta-blockers – between 45 and 85%; power of 80%; and type-I error of 5% is given in Table 1. With these assumptions our recruitment target is 500 patients with COPD and MI who are discharged alive. Assuming in-hospital mortality of 12.5% (28) we plan to consent 571 patients to achieve this goal.

© 2024 LaFon DC et al. *JAMA Network Open*.

Table 1: Estimated sample size assuming power of 80%, α=0.05, and risk of death or re-hospitalization at 6 months of 25% in group receiving beta blockers.

Risk in group not receiving bet a blockers	Proportion of patients discharged on Beta-blockers										
	0.4	0.45	0.5	0.55	0.6	0.65	0.7	0.75	0.8	0.85	0.9
0.3	2528	2473	2471	2519	2622	2791	3053	3451	4083	5174	7401
0.325	1139	1119	1122	1148	1200	1283	1409	1600	1901	2420	3478
0.35	648	638	642	660	692	743	819	934	1115	1426	2058
0.375	418	413	417	430	453	488	540	618	741	951	1379

5. **Data collection and handling.** Data will be collected on study-specific case report forms (CRF) at each center and then data entered to the web-based system housed at the Data Coordinating Center (DCC) at the University of Minnesota.
    a. **Study Confidentiality and Privacy.** Study participants are identified by a unique number with a check digit for data integrity and an enrollment code consisting of the first letter of the participant's last name and the last two digits of their birth year. No PHI will be collected on study CRFs or transmitted to the DCC. Any identifying information that could be classified as PHI will be kept separate from the participant CRFs in a secure environment at the clinical center accessible only to study staff allowed access to PHI. Documents which contain PHI requested by the DCC will be properly de-identified prior to transmission so that no PHI can be obtained from the records.
    b. **Data Security.** The web-based data submission software is Oracle Application Server 11g Release 1. All data transmitted from the clinical sites to the DCC and from the DCC to the clinical sites is SSL encrypted. The cryptographic libraries used for SSL are designed to meet FIPS 140-2 Level 2 certification. The website is secured by individual usernames and strong passwords. Non-secure ports of all servers are located behind the DCC network firewall and accessible only under the following conditions: 1) a workstation physically present in the building and physically connected to the network 2) granted system access to the database server 3) a user name and strong password for that database server 4) a second username and strong password for database software 5) granted database access to data items. All non-essential ports of the database server and web servers are closed. Only one secure (HTTPS) port on the website server is open outside of the firewall; this port displays the login link to the secured website.

       Clinical site staff may access individual data items for a given participant (only those in their own clinic) for review, but they are unable to download any participant data, aggregate it, cross reference an individual's data, or view participant data from another clinical site via the website.

       All servers are physically located in a secured room which is accessible only by a key card; currently, only two system administrators, one financial administrator, and the Database Administrator have access to this room. In addition, the secured room is located in a secured suite which is also accessible only by key cards given to current University of Minnesota employees located in the suite. The building is locked and accessible only by University of Minnesota employees located in the building after business hours and on weekends.

       The security administrator for Oracle RDBMS and Oracle Application Server is the Database Administrator. A network system administrator regularly monitors for occurrences of attempted access to the network by unauthorized users. Only the Database Administrator has access to sensitive software passwords. On-site backup of study data is performed nightly; off-site backups are taken twice weekly and stored at a secure University of Minnesota location, in a locked office in a secure suite. Additional redundancy is accomplished by storing duplicate database data files and control files on a separate server for disaster mitigation.

© 2024 LaFon DC et al. *JAMA Network Open*.

6. **Monitoring Plan:** Monitoring to ensure the study proceeds in compliance with the protocol is multifaceted including real time oversight by the local PIs and regular and real time monitoring of entered clinical data by staff at DCC as well as by an independent Observational Study Monitoring Board (OSMB). The OSMB will be made up of at least one cardiologist, one pulmonologist and a statistician and will meet every six months by teleconference. The responsibilities of the OSMB are to track performance of individual centers (including possible recommendations on actions to be taken regarding any center that performs unsatisfactorily); monitor overall recruitment, quality control, and data analysis; and oversee the overall scientific direction of the study. The DCC will prepare materials for the OSMB and will schedule meetings and prepare draft agendas. The DCC will also conduct monthly teleconferences throughout the study to review study enrolment and retention, procedures, adherence to protocol, and timeliness of data entry.

7. **Human subjects protection**
    a. Protection against risks. The procedures involved in this study are limited to data collection using questionnaires, review of EMR, and phone calls. Risks are limited to risks related to invasion of privacy and breach of confidentiality and are no greater than minimal. To protect against these risks, informed consent and will be obtained and filled out in private rooms and study visits will be conducted in private rooms (Option 1 ony). All data will be stored on password-protected computers with access limited to authorized study personnel and adequate measures will be taken to maintain patient confidentiality at all times. All data collected from subjects will be labelled with a unique code that has no individually identifiable information. Patient information will be maintained as confidential to the extent permitted by law. The PI and other designated study staff (all who have been educated/trained on the protection of human subjects in research) will have access to identifiable private information of subjects, which may also be shared if requested with the UAB IRB, DOD, and others who are responsible for ensuring compliance with laws and regulations related to research, including OHRP. All patient identifiers will be removed prior to any data analyses, or summary of data for publication in peer-reviewed journals.

    b. Potential benefits. There are no direct benefits to subjects who choose to participate in this observational study. Participants may have the satisfaction of participating in a research study that will potentially lead to improved treatment of COPD. The risks involved in the protocol are minimal, the potential benefits to our understanding of the overall risk-benefit of beta-blockers in COPD patients with acute MI are significant, and thus the overall risk-benefit favors the conduct of the study.

    c. Importance of knowledge gained. The results of proposed Aim 1 will provide data about the prevalence and clinical characteristics of COPD in the hospitalized population with MI in our network. The Aim will also provide an estimate of the number of annual admissions for patients with COPD and MI. Aim 2 will provide valuable information about the associations between beta-blocker use at discharge and the risk for all-cause mortality, recurrent ischemic events, and hospitalization for COPD exacerbation adjusting for baseline characteristics and COPD severity. Even though the parent BLOCK-COPD trial strongly indicated that patients with COPD and at risk for exacerbation should not be prescribed beta-blockers in the absence of an established indication for the drugs such as recent myocardial infarction or heart failure, questions remain about their use in the latter populations. The results of our proposed observational study would provide more robust and contemporary data regarding the overall risk-benefit of beta-blockers in COPD patients with acute MI and provide preliminary data about the feasibility of recruitment for a subsequent randomized trial.

© 2024 LaFon DC et al. *JAMA Network Open*.

eTable 1. Assessment of the proportional hazards assumption- test for independence between the Schoenfeld residuals and time for each model.

	**Unadjusted model[a]**	**Adjusted model[b]**	**IPTW model[a]**
Death, hospitalization or revascularization (primary)	0.06	Average: 0.03[c]<br>%<0.05: 100%	0.04
CVD death or CVD-related hospitalization/revascularization (secondary)	0.56	Average:0.14<br>%<0.05: 0%	0.51
Respiratory/COPD-related death or hospitalization (secondary)	0.83	Average:0.44<br>%<0.05: 0%	0.82

[a] Score test is equivalent to the log rank test which does not rely on the proportional hazards assumption being satisfied

[b] Results presented are the average p-value and percentage of p-values <0.05 across the 30 imputed data sets.

[c] Given that the proportional hazards assumption did not hold, the estimated hazard ratio for this model can be considered as the weighted average across time.

© 2024 LaFon DC et al. *JAMA Network Open*.

eTable 2. Standardized mean differences (SMD) for comparison of demographics, medical history, and clinical characteristics between individuals with COPD with follow-up information who were and were not discharged with a prescription for beta blockers.

**Characteristics**	SMD
**Demographics**	
Age[a]	0.094
Sex[a]	0.029
Clinic	0.662
Race	0.043
Ethnicity	0.095
Body mass index (BMI)[a]	0.110
Smoking status [a]	0.103
**Medical History**	
High blood pressure [a]	0.102
Hyperlipidemia	0.019
Coronary artery disease [a]	0.276
Heart attack	0.060
Heart failure	0.085
Prior percutaneous coronary intervention [a]	0.201
Prior coronary artery bypass grafting	0.012
Peripheral vascular disease	0.053
Stroke [a]	0.278
Diabetes mellitus	0.025
Cirrhosis	0.033
Asthma	0.072
Obstructive sleep apnea	0.085
End stage renal disease	0.016
Depression[a]	0.204
Anxiety [a]	0.329
Cancer [a]	0.107
Organ transplantation[a]	0.162
**Treatments/characteristics during admission**	
Thrombolytics[a]	0.197
Percutaneous coronary intervention [a]	0.061
Coronary artery bypass grafting [a]	0.253
Experienced cariogenic shock [a]	0.344
Diagnosis for current admission	0.068
Ejection fraction [a]	0.051
Admitted to ICU	0.006
Days in ICU [a]	0.306
Intubated	0.060

© 2024 LaFon DC et al. *JAMA Network Open*.

Characteristics	SMD
**Demographics**	
Discharge disposition [a]	0.122
Days in hospital [a]	0.204
Discharged on hospice	0.146
**COPD Characteristics**	
Pre- or Post-Bronchodilator spirometry values [a]	0.122
FEV1[b]	0.149
FEV1 % predicted [a]	0.144
FVC	0.002
FVC % predicted [a]	0.149
Prescription for home oxygen [a]	0.150
Received a course of systemic corticosteroids and/or antibiotics in past year	0.134
Number of courses [a]	0.043
Respiratory episode required care in the ED	0.026
Respiratory episode led to hospitalization [a]	0.150
Number of hospitalizations in past year	0.097
Respiratory episode required intubation [a]	0.130

[a]Variables included in model for calculating propensity score

[b] Even though the SMD was >0.10, this variable was not chosen due to potential multicolinarity with FEV1 % predicted

© 2024 LaFon DC et al. *JAMA Network Open*.

eTable 3. Number of individuals who experienced each event type by beta blocker prescription at discharge.

	Not discharged with beta blockers (N=73)	Discharged with beta blockers (N=480)
**Primary outcome**		
Death, hospitalization, or revascularization	35 (47.9%)	195 (40.6%)
**Secondary outcomes**		
CVD-related death/hospitalization or revascularization	20 (27.0%)	114 (24%)
Respiratory/COPD-related death or hospitalization	10 (13.7%)	45 (9.4%)
**Nonfatal events**		
Overall hospitalization or revascularization	29 (39.7%)	163 (34.0%)
CVD hospitalization or revascularization	17 (23.3%)	105 (21.9%)
COPD or respiratory hospitalization	9 (12.3%)	37 (7.7%)
Antibiotics or steroids for exacerbation	14 (19.2%)	82 (17.1%)

© 2024 LaFon DC et al. *JAMA Network Open*.

eReferences

eMethods

1. Rubin DB. Multiple Imputation for Nonresponse in Surveys. New York: John Wiley and Sons; 1987. P.76

eAppendix

1. Collaborators GBDCoD. Global, regional, and national age-sex-specific mortality for 282 causes of death in 195 countries and territories, 1980-2017: a systematic analysis for the Global Burden of Disease Study 2017. Lancet. 2018;392(10159):1736-88.
2. Vogelmeier CF, Criner GJ, Martinez FJ, Anzueto A, Barnes PJ, Bourbeau J, et al. Global Strategy for the Diagnosis, Management, and Prevention of Chronic Obstructive Lung Disease 2017 Report. GOLD Executive Summary. American journal of respiratory and critical care medicine. 2017;195(5):557-82.
3. Bhatt SP, Dransfield MT. Chronic obstructive pulmonary disease and cardiovascular disease. Translational research : the journal of laboratory and clinical medicine. 2013;162(4):237-51.
4. Chen W, Thomas J, Sadatsafavi M, FitzGerald JM. Risk of cardiovascular comorbidity in patients with chronic obstructive pulmonary disease: a systematic review and meta-analysis. The Lancet Respiratory medicine. 2015;3(8):631-9.
5. Westerik JA, Metting EI, van Boven JF, Tiersma W, Kocks JW, Schermer TR. Associations between chronic comorbidity and exacerbation risk in primary care patients with COPD. Respiratory research. 2017;18(1):31.
6. Niewoehner DE, Lokhnygina Y, Rice K, Kuschner WG, Sharafkhaneh A, Sarosi GA, et al. Risk indexes for exacerbations and hospitalizations due to COPD. Chest. 2007;131(1):20-8.
7. Ai-Ping C, Lee KH, Lim TK. In-hospital and 5-year mortality of patients treated in the ICU for acute exacerbation of COPD: a retrospective study. Chest. 2005;128(2):518-24.
8. Dransfield MT, Rowe SM, Johnson JE, Bailey WC, Gerald LB. Use of beta blockers and the risk of death in hospitalised patients with acute exacerbations of COPD. Thorax. 2008;63(4):301-5.
9. Divo M, Cote C, de Torres JP, Casanova C, Marin JM, Pinto-Plata V, et al. Comorbidities and risk of mortality in patients with chronic obstructive pulmonary disease. American journal of respiratory and critical care medicine. 2012;186(2):155-61.
10. Miller J, Edwards LD, Agusti A, Bakke P, Calverley PM, Celli B, et al. Comorbidity, systemic inflammation and outcomes in the ECLIPSE cohort. Respir Med. 2013;107(9):1376-84.
11. Hjalmarson A, Elmfeldt D, Herlitz J, Holmberg S, Malek I, Nyberg G, et al. Effect on mortality of metoprolol in acute myocardial infarction. A double-blind randomised trial. Lancet. 1981;2(8251):823-7.
12. Hjalmarson A, Goldstein S, Fagerberg B, Wedel H, Waagstein F, Kjekshus J, et al. Effects of controlled-release metoprolol on total mortality, hospitalizations, and well-being in patients with heart failure: the Metoprolol CR/XL Randomized Intervention Trial in congestive heart failure (MERIT-HF). MERIT-HF Study Group. JAMA : the journal of the American Medical Association. 2000;283(10):1295-302.
13. Quint JK, Herrett E, Bhaskaran K, Timmis A, Hemingway H, Wedzicha JA, et al. Effect of beta blockers on mortality after myocardial infarction in adults with COPD: population based cohort study of UK electronic healthcare records. Bmj. 2013;347:f6650.
14. Egred M, Shaw S, Mohammad B, Waitt P, Rodrigues E. Under-use of beta-blockers in patients with ischaemic heart disease and concomitant chronic obstructive pulmonary disease. Qjm. 2005;98(7):493-7.
15. Gottlieb SS, McCarter RJ, Vogel RA. Effect of beta-blockade on mortality among high-risk and low-risk patients after myocardial infarction. The New England journal of medicine. 1998;339(8):489-97.
16. Su TH, Chang SH, Kuo CF, Liu PH, Chan YL. beta-blockers after acute myocardial infarction in patients with chronic obstructive pulmonary disease: A nationwide population-based observational study. PloS one. 2019;14(3):e0213187.
17. Bhatt SP, Wells JM, Kinney GL, Washko GR, Jr., Budoff M, Kim YI, et al. beta-Blockers are associated with a reduction in COPD exacerbations. Thorax. 2016;71(1):8-14.
18. Etminan M, Jafari S, Carleton B, FitzGerald JM. Beta-blocker use and COPD mortality: a systematic review and meta-analysis. BMC pulmonary medicine. 2012;12:48.
19. Du Q, Sun Y, Ding N, Lu L, Chen Y. Beta-blockers reduced the risk of mortality and exacerbation in patients with COPD: a meta-analysis of observational studies. PloS one. 2014;9(11):e113048.

© 2024 LaFon DC et al. *JAMA Network Open*.

20. Mancini GB, Etminan M, Zhang B, Levesque LE, FitzGerald JM, Brophy JM. Reduction of morbidity and mortality by statins, angiotensin-converting enzyme inhibitors, and angiotensin receptor blockers in patients with chronic obstructive pulmonary disease. Journal of the American College of Cardiology. 2006;47(12):2554-60.
21. Suissa S, Ernst P. Beta-Blockers in COPD: A Methodological Review of the Observational Studies. COPD. 2018;15(5):520-5.
22. Bhatt SP, Connett JE, Voelker H, Lindberg SM, Westfall E, Wells JM, et al. beta-Blockers for the prevention of acute exacerbations of chronic obstructive pulmonary disease (betaLOCK COPD): a randomised controlled study protocol. BMJ Open. 2016;6(6):e012292.
23. Dransfield MT, Voelker H, Bhatt SP, Brenner K, Casaburi R, Come CE, et al. Metoprolol for the Prevention of Acute Exacerbations of COPD. The New England journal of medicine. 2019.
24. Chen J, Radford MJ, Wang Y, Marciniak TA, Krumholz HM. Effectiveness of beta-blocker therapy after acute myocardial infarction in elderly patients with chronic obstructive pulmonary disease or asthma. Journal of the American College of Cardiology. 2001;37(7):1950-6.
25. Metoprolol in acute myocardial infarction. Patients and methods. The MIAMI Trial Research Group. The American journal of cardiology. 1985;56(14):3G-9G.
26. Dodson JA, Hajduk AM, Murphy TE, Geda M, Krumholz HM, Tsang S, et al. Thirty-Day Readmission Risk Model for Older Adults Hospitalized With Acute Myocardial Infarction. Circ Cardiovasc Qual Outcomes. 2019;12(5):e005320.
27. Hall M, Dondo TB, Yan AT, Mamas MA, Timmis AD, Deanfield JE, et al. Multimorbidity and survival for patients with acute myocardial infarction in England and Wales: Latent class analysis of a nationwide population-based cohort. PLoS Med. 2018;15(3):e1002501.
28. Agarwal M, Agrawal S, Garg L, Garg A, Bhatia N, Kadaria D, et al. Effect of Chronic Obstructive Pulmonary Disease on In-Hospital Mortality and Clinical Outcomes After ST-Segment Elevation Myocardial Infarction. The American journal of cardiology. 2017;119(10):1555-9.
29. Rothnie KJ, Yan R, Smeeth L, Quint JK. Risk of myocardial infarction (MI) and death following MI in people with chronic obstructive pulmonary disease (COPD): a systematic review and meta-analysis. BMJ Open. 2015;5(9):e007824.
30. Andell P, Koul S, Martinsson A, Sundstrom J, Jernberg T, Smith JG, et al. Impact of chronic obstructive pulmonary disease on morbidity and mortality after myocardial infarction. Open Heart. 2014;1(1):e000002.
31. Enriquez JR, de Lemos JA, Parikh SV, Peng SA, Spertus JA, Holper EM, et al. Association of chronic lung disease with treatments and outcomes patients with acute myocardial infarction. Am Heart J. 2013;165(1):43-9.
32. Rothnie KJ, Quint JK. Chronic obstructive pulmonary disease and acute myocardial infarction: effects on presentation, management, and outcomes. Eur Heart J Qual Care Clin Outcomes. 2016;2(2):81-90.
33. Campo G, Guastaroba P, Marzocchi A, Santarelli A, Varani E, Vignali L, et al. Impact of COPD on long-term outcome after ST-segment elevation myocardial infarction receiving primary percutaneous coronary intervention. Chest. 2013;144(3):750-7.
34. Andell P, Erlinge D, Smith JG, Sundstrom J, Lindahl B, James S, et al. beta-blocker use and mortality in COPD patients after myocardial infarction: a Swedish nationwide observational study. J Am Heart Assoc. 2015;4(4).
35. Dunlay SM, Weston SA, Killian JM, Bell MR, Jaffe AS, Roger VL. Thirty-day rehospitalizations after acute myocardial infarction: a cohort study. Annals of internal medicine. 2012;157(1):11-8.

© 2024 LaFon DC et al. *JAMA Network Open*.
