# Supplementary material for: β-Blocker Use and Clinical Outcomes in Patients With COPD Following Acute Myocardial Infarction
Source: JAMA Netw Open. 2024 May 21;7(5):e247535. doi: 10.1001/jamanetworkopen.2024.7535 (PMC11109775; doi:10.1001/jamanetworkopen.2024.7535)
Supplement: Supplement 2. — Data Sharing Statement [file jamanetwopen-e247535-s002.pdf]

Data Sharing Statement

LaFon. β-Blocker Use and Clinical Outcomes in Patients With COPD Following Acute Myocardial Infarction. *JAMA Netw Open*. Published May 21, 2024.
doi:10.1001/jamanetworkopen.2024.7535

Data
Data available: No

Additional Information
Explanation for why data not available: The study is registered on clinicaltrials.gov (NCT04717492).
